# In Silico Prediction, Molecular Docking and Dynamics Studies of Steroidal Alkaloids of *Holarrhena pubescens* Wall. ex G. Don to Guanylyl Cyclase C: Implications in Designing of Novel Antidiarrheal Therapeutic Strategies

**DOI:** 10.3390/molecules26144147

**Published:** 2021-07-08

**Authors:** Neha Gupta, Saurav Kumar Choudhary, Neeta Bhagat, Muthusamy Karthikeyan, Archana Chaturvedi

**Affiliations:** 1 Centre for Medical Biotechnology, Amity Institute of Biotechnology, Amity University Uttar Pradesh, Noida 201313, India; neha16@bu.edu (N.G.); saurav20216@iiitd.ac.in (S.K.C.); nbhagat@amity.edu (N.B.); 2 Pharmacogenomics and CADD Laboratory, Department of Bioinformatics, Alagappa University, Karaikudi, Tamil Nadu 630004, India

**Keywords:** diarrhea, enterotoxigenic *E. coli* (ETEC), extracellular domain (ECD) of GC-C, guanylyl cyclase c (GC-C), heat stable enterotoxin (STa), steroidal alkaloids

## Abstract

The binding of heat stable enterotoxin (STa) secreted by enterotoxigenic *Escherichia coli* (ETEC) to the extracellular domain of guanylyl cyclase c (ECD_GC-C_) causes activation of a signaling cascade, which ultimately results in watery diarrhea. We carried out this study with the objective of finding ligands that would interfere with the binding of STa on ECD_GC-C_. With this view in mind, we tested the biological activity of a alkaloid rich fraction of *Holarrhena pubescens* against ETEC under in vitro conditions. Since this fraction showed significant antibacterial activity against ETEC, we decided to test the screen binding affinity of nine compounds of steroidal alkaloid type from *Holarrhena pubescens* against extracellular domain (ECD) by molecular docking and identified three compounds with significant binding energy. Molecular dynamics simulations were performed for all the three lead compounds to establish the stability of their interaction with the target protein. Pharmacokinetics and toxicity profiling of these leads demonstrated that they possessed good drug-like properties. Furthermore, the ability of these leads to inhibit the binding of STa to ECD was evaluated. This was first done by identifying amino acid residues of ECD_GC-C_ binding to STa by protein–protein docking. The results were matched with our molecular docking results. We report here that holadysenterine, one of the lead compounds that showed a strong affinity for the amino acid residues on ECD_GC-C_, also binds to STa. This suggests that holadysenterine has the potential to inhibit binding of STa on ECD and can be considered for future study, involving its validation through in vitro assays and animal model studies.

## 1. Introduction

Diarrhea is a major public health problem in rural parts of India. The disease is usually transmitted by the contamination of drinking water and foods with fecal matter. It is responsible for the morbidity and infant mortality prevalent in areas with poor sanitation and crowded conditions [1,2,3]. The disease is a gastrointestinal disorder, characterized by an increase in stool frequency and a change in its consistency [4]. One of the important etiological agents for intestinal infection in humans has been reported to be enterotoxigenic *E. coli* (ETEC). This micro-organism has also been reported to be associated with traveler’s diarrhea [5].

ETEC, a very diverse group of pathogenic *E. coli*, colonizes the small intestine and produces heat-stable enterotoxin (STa) [6]. The virulence of ETEC is believed to be associated with heat-stable enterotoxin (STa). It disrupts intestinal fluid homeostasis and promotes the hypersecretion of fluid and electrolytes through the activation of guanylyl cyclase c in small intestine mucosal cells [7]. The guanylyl cyclase c (GC-C) is a member of the guanylyl cyclase-coupled receptors family (GCs) [8]. It is a multi-domain receptor with an extracellular ligand binding domain (ECD) at the N-terminal end and an intracellular domain at the C terminal end [9,10]. The extracellular domain followed by a transmembrane domain is attached to the catalytic domain through a linker region [11]. The topological organization of guanylyl cyclase c shares a similarity with the receptor proteins (NPR-A and NPR-B) for atrial and brain natriuretic peptides, respectively [12]. 

Binding of the paracrine hormone, such as guanylin or uroguanylin, to the extracellular domain (ECD) elicits a conformational change that increases GC-C activity, resulting in an elevation of cGMP level [10]. This in turn causes protein kinase mediated activation of cystic fibrosis transmembrane conductance regulator (CFTR), which is an apical ion channel responsible for the efflux of chloride ions [10]. Heat stable enterotoxin (STa), which will be referred to as STa in this study, is an agonist of GC-C [13,14]. It is an 18-amino acid long peptide, with three-disulfide bridges [15]. The binding of STa to the extracellular receptor domain of guanylyl cyclase c (ECD_GC-C_), located at the luminal membrane of intestinal epithelial cells, induces a several-fold higher intracellular production of cGMP than that of guanylin and uroguanylin [16]. This leads to the excessive secretion of chloride and bicarbonate ions and inhibition of Na^+^/H^+^ exchanger isotype 3 (NHE3), resulting in reduced absorption of Na^+^ [17]. Thus, GC-C signaling plays a pivotal role in the regulation of intestinal fluid and electrolyte homeostasis. In addition, GC-C also protects the intestinal mucosal barrier by regulating myosin light chain kinase (MLCK) activity and tight junction (TJ) assembly [18]. It has been shown that dysregulation in intestinal barrier function can cause several intestinal diseases such as inflammatory bowel disease (IBD) and irritable bowel syndrome (IBS).

The methods, which are currently used for the management of secretory diarrhea, are limited to the use of oral rehydration therapy aimed at replenishing the body with salt and water to prevent dehydration [19]. Unfortunately, specific therapeutic options for its treatment are mostly unavailable. The drugs available for the treatment of diarrhea, such as loperamide, are associated with various side effects, such as abdominal discomfort, lethargy, respiratory depression, and coma, which outweigh its benefits in reducing stool frequency [20,21]. As an alternative solution to this problem, the World Health Organization (WHO) has initiated a diarrhea disease control program. The program emphasizes the need to explore documented traditional medicinal knowledge and indigenous herbal preparations [22]. The biggest advantage of exploring these traditional databases is that these drugs have already been tested for thousands of years and their clinical observations are already available [23]. The Indian System of Medicine through Ayurveda describes many plant-based drugs for the effective treatment of diarrhea. One of the very popular drugs from the Ayurvedic database for diarrhea treatment is *Holarrhena pubescens* Wall. ex G. Don (kutaj). It is a deciduous tree, which grows in the Himalayan region and distributed throughout India. The stem bark of *Holarrhena pubescens* (kutaj) is useful for the treatment of diarrhea and dysentery [24]. Ethanolic extract of *H. antidysenterica* seeds controls diarrhea and decreases the severity of the clinical signs of castor oil and *E. coli* induced diarrhea in Wistar rats [25]. Seed and bark extracts of the plant have demonstrated their ability to kill free-living *Entamoeba histolytica* in the dysenteric stool of experimentally infected kittens [26]. With this background in mind, *Holarrhena pubescens* (kutaj) was identified as the drug of choice for the current study.

It has been observed that any kind of disruption in the normal functioning of guanylyl cyclase c signaling can influence the maintenance of the intestinal barrier, as well as causing inhibition of inflammation, visceral pain signaling and tumorigenesis, abdominal pain, constipation, and, of course, diarrhea [27,28]. For this reason, GC-C has been selected as an important target in developing therapies for various gastrointestinal diseases, such as functional gastrointestinal disorders and IBDs [29]. The present study focuses on testing the potential of steroidal alkaloids of *Holarrhena pubescens* Wall. ex G. Don (kutaj) for blocking GC-C during ETEC induced diarrhea. The study was conducted using an in silico approach.

## 2. Results and Discussion

### 2.1. Antimicrobial Activity

The antibacterial activity of *Holarrhena pubescens* (kutaj) was tested against diarrhea causing clinical isolates of Enterotoxigenic *E. coli* (ETEC). To accomplish this, an alkaloid-rich fraction, prepared from the stem bark of *Holarrhena pubescens* (kutaj), was tested against ETEC at a dose of 100 mg/mL. The results given in Table 1 demonstrate a 16 mm zone of inhibition, representing significant antimicrobial activity against the tested strains. The effectiveness of the alkaloid fraction was determined by measuring the minimum inhibitory concentration. The MIC for the tested strain was found to be 50 mg/mL. In the case of the positive control, gentamycin, the zone of inhibition was 35 mm (Table 1). These results are in agreement with those of Voravuthikunchai et al. (2004), where they demonstrated the antibacterial activity of *Holarrhena pubescens* against diarrheagenic *E. coli* 0157:H7 [30]. These results point towards the antibacterial activity of the alkaloids present in the drug sample.

There have been reports showing that some piperidine type alkaloids, such as N-2-(propylamino)-6-phenylpyrimidin-4-one–substituted piperidines derivative, blocked the STa induced chloride secretory response in animal models [31]. The stem bark of *Holarrhena pubescens* has been reported to be rich in therapeutically important steroidal alkaloids [32]. In the next step we screened nine steroidal alkaloids of *Holarrhena pubescens* (kutaj) for their binding affinity towards ECD_GC-C_ using an in silico approach.

### 2.2. Sequence Analysis and Model Generation

**Figure 1:**
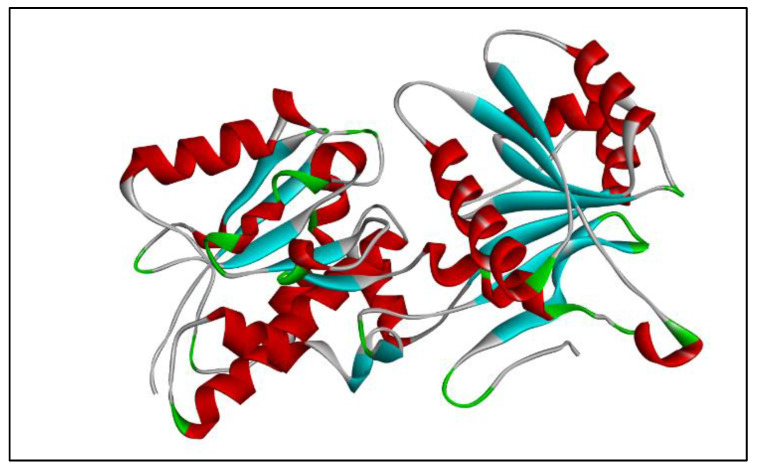
3D model of ECD generated by SWISS MODEL workspace.

Since the crystal structure of GC-C protein is not available in RCSB PDB and SCOP, its 3D model was built using SWISS MODEL workspace [33]. Guanylyl cyclase c has been reported to be a 1073 amino acid long sequence [9,34]. For the model generation the sequence corresponding to the extracellular domain (ECD) of the GC-C receptor (with UniProt/NCBI accession number P25092) was used as a query sequence for a PSI-BLAST search in the PDB database. The query sequence was 407 amino acids long, ranging from 24–430 amino acids of the full length receptor of guanylyl cyclase c (GC-C). The search resulted in three templates belonging to Natriuretic Peptide Receptor-C (NPR-C) (1JDN, 1YK0 and 1YK1). All three templates showed the same percentage identity (22.29%) with the query sequence. This is in agreement with a previous report which showed that the ECD of Natriuretic Peptide Receptor-C (NPR-C) shares about 20% of its sequence with that of GC-C [35]. The template search was also carried out in the SWISS MODEL database using the same query sequence of extracellular domain (ECD). This search gave rise to 50 templates, out of which 1YK1-A and 1YK1-B, belonging to the chain A and chain B of Natriuretic Peptide Receptor-C (NPR-C), showed the maximum percentage identity with the ECD of GC-C. In the next step 1YK1-A was selected for modeling since it appeared in both the searches, and a homology model for ECD was built based on the structure of chain A of NPR-C. The natriuretic peptide receptor- C (NPR-C) is not a guanylyl cyclase but is homologous to NPR-A, which happens to be a GC family member [36]. The structure of NPR-C in ligand bound form and unbound form is available [37,38]. Analysis of the available crystal structure of the ligand bound extracellular domain (ECD) of NPR-C and NPR-A receptors demonstrated that even though the sequence homology between them was low (less than 30%), their structures were remarkably similar [39]. Based on this analysis 1YK1-A was used for model generation. The generated model of ECD is presented as Figure 1.

### 2.3. Validation of Homology Model

**Figure 2:**
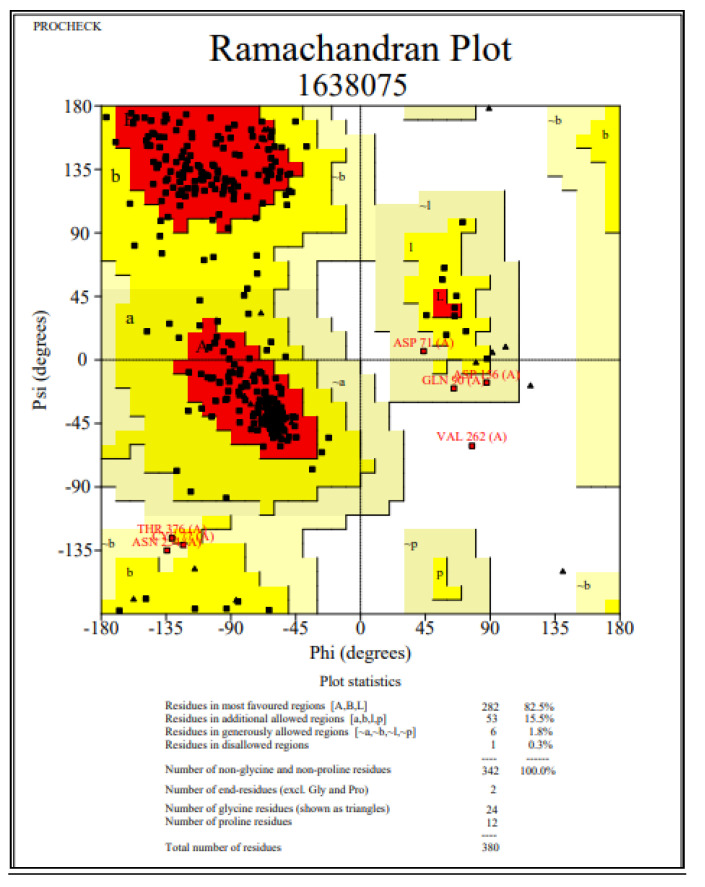
Ramachandran plot of ECD obtained through PROCHECK.

The quality of the ECD model was assessed using various tools. The stereo chemical quality and accuracy of the model was tested using the PROCHECK server [40]. The results from PROCHECK have been reported as a Ramachandran plot (Figure 2). A structure with >90% of its residues in the most favored region of the Ramachandran plot is considered to be as accurate as a 2 Å resolution structure. The statistics for the current model of ECD showed 82.5% amino acid residues in the core region, 15.5% in the allowed region, 1.8% in the generously allowed region, and 0.3% in the disallowed region (Figure 2). The score for the backbone conformation was normal, with a slight deviation. 

**Figure 3:**
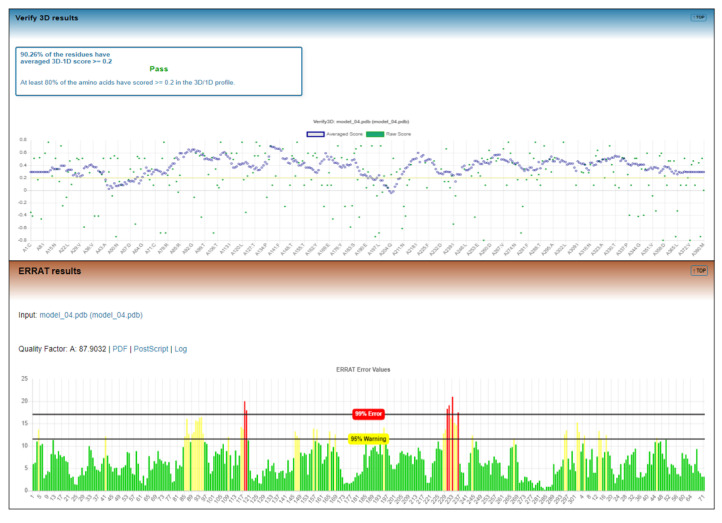
Verify 3D and ERRAT Plot.

For further analysis of the generated model of ECD, ERRAT [41] and Verify3D [42] were used. Verification by VERIFY3D showed a score of 90.26%, and none of the amino acids had a negative score (Figure 3). A compatibility score above zero is an indication of an acceptable side chain environment [42]. The overall quality factor predicted by the ERRAT server for the current model was 87.90 (Figure 3). The generally accepted range for a high-quality model is ≥50 [41]. This indicates that the backbone conformation and non-bonded interactions of the generated model fit well within the range of a high-quality model. 

### 2.4. Active Site Prediction 

**Figure 4:**
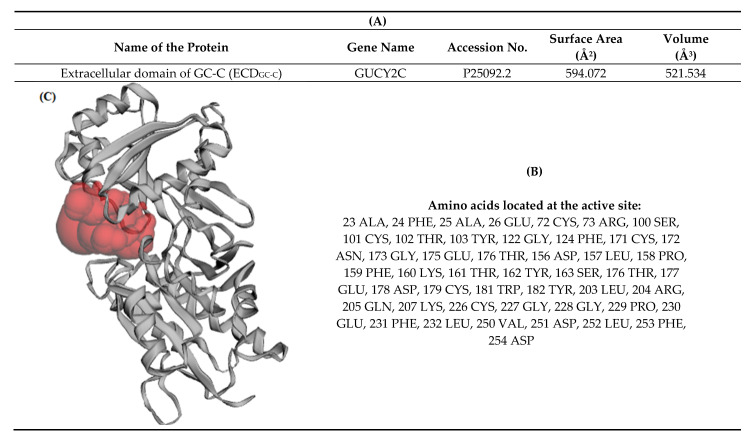
Active site prediction of extracellular domain (ECD) through CASTp. (**A**) Area and volume of the active site. (**B**) Amino acids located at the active site. (**C**) 3D structure of the active site.

The active site of the generated model of ECD was predicted using the CASTp3.0 server [43]. The volume of the pocket was found to be 521.534 Å^3^ and the surface area of the pocket was 594.072 Å^2^ (Figure 4).

### 2.5. Docking Study

Ligands belonging to the class of steroidal alkaloids from *Holarrhena pubescens* were docked to the active site of ECD_GC-C_ using AutoDock4.2 [44]. The binding energies observed for various test ligands, such as pubescine, holadysenterine, holanamine, kurchessine, holadienine, conessimine, conessine, isoconessimine, kurchine, and loperamide, were in the following order −8.05, −8.06, −8.44, −8.52, −8.54, −8.59, −9.00, −9.05, −9.05, and −8.05 kcal/mol, respectively (Table 2). Amongst all the ligands, pubescine and loperamide had the highest binding energy of −8.05 kcal/mol. On the other hand, isoconessimine and kurchine had the lowest energy (−9.05 kcal/mol) (Table 2).

**Figure 5:**
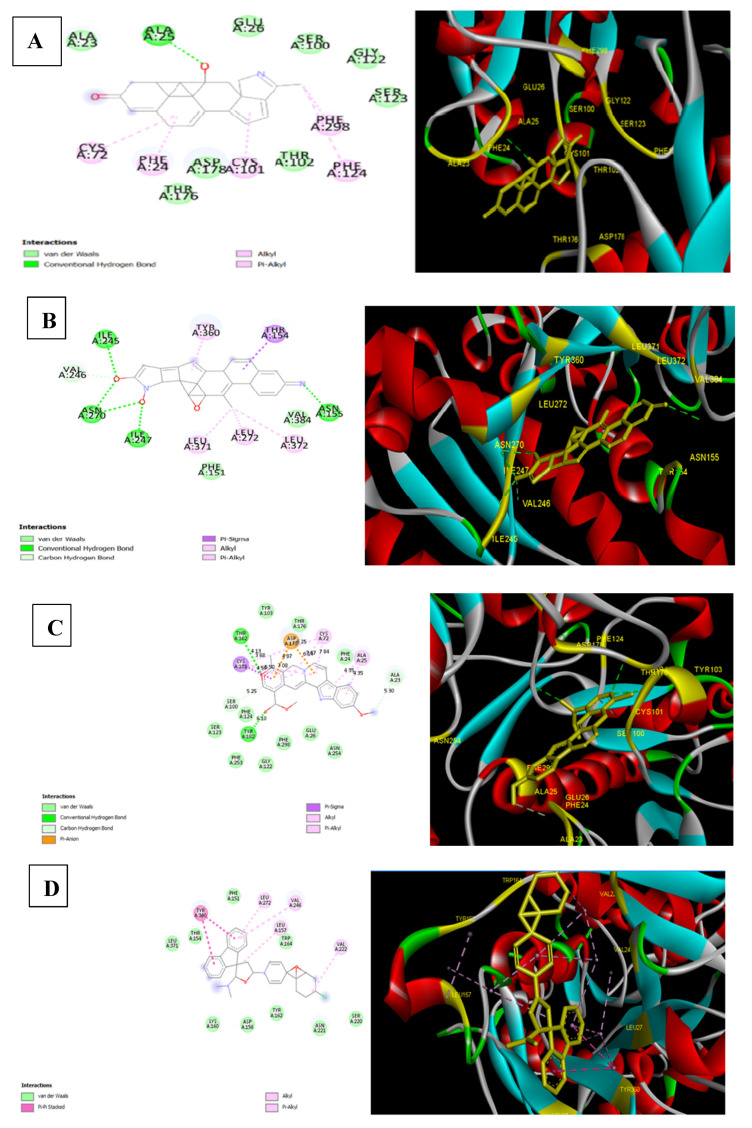
Visualization of the interaction between the ligands selected as lead compounds and the ECD and Hydrogen bond detection using Discovery Studio. (**A**) Holanamine; (**B**) Holadysenterine; (**C**) Pubescine; (**D**) Control Drug-Loperamide.

Binding energy is a function of the stability of the complex formed between ligand and target protein. It also optimizes new bonds that in turn may affect the biological activity of the resulting complex. To further display various interactions involved between ligands and target protein at the active site, the docked complexes were visualized through Discovery Studio Visualizer [45]. Our results demonstrated that only the docking of holanamine, pubescine, and holadysenterine displayed the formation of hydrogen bonds with the receptor protein (Figure 5). In case of holanamine, one hydrogen bond with ALA25 of the active site of ECD was observed (Figure 5). On the other hand, pubescine displayed two hydrogen bonds with the receptor (Figure 5). Amino acids engaged in hydrogen bond formation with pubescine included THR102 and TYR182 (Figure 5). The docked complex of holadysenterine and target protein formed five hydrogen bonds with ASN155, ILE245, ILE247, and ASN270 (Figure 5). Interestingly, the ASN270 of ECD formed two hydrogen bonds with holadysenterine. The importance of hydrogen bonds for the binding affinity of the target drug has been described extensively by Patil et al. (2010) [46]. The docked complexes of all three ligands, viz pubescine, holadysenterine, and holanamine, also displayed van der Waals, pi-sigma, pi-alkyl, and alkyl type interactions (Figure 5).

The docking results obtained with the test ligands were compared with loperamide, a commercially available drug for the treatment of diarrhea. The docking of the drug against ECD showed a binding energy of −8.05 kcal/mol (Figure 5). This interaction was achieved by van der Waals forces, pi-pi stacking, pi-alkyl, and alkyl interactions, which probably helped loperamide to intercalate at the binding site of ECD. But these are weaker interactions in comparison to the hydrogen bonds [47]. In fact, amongst all the intermolecular non-covalent interactions, hydrogen bonds play a central role in the binding of a ligand to the active site of the protein. In this context the docked complex of loperamide is probably not as stable as that of the complexes formed by pubescine, holadysenterine, and holanamine.

These results suggest that ECD_GC-C_ is not a suitable binding site for loperamide. As a matter of fact, loperamide reduces gut motility by acting on peripheral opioid receptors [48].

Based on the results obtained from the molecular docking study, holanamine, holadysenterine, and pubescine were identified as the best hits and taken up for further study.

### 2.6. Drug-Likeness Prediction 

The test ligands were assessed for their drug-like properties based on Lipinski’s rule of five [49] and ADMET properties. Logically, these ligands should not be subject to the issue of bioavailability, as they are targeted towards GC-C, which is expressed on the luminal side of the intestinal epithelium [50]. However, it needs to be highlighted here that these test ligands are small molecular weight natural compounds and have not been designed to be impermeable to the membrane. Therefore, either as parent compounds or as metabolites, they are likely to have at least some absorption by the systemic compartments and subsequently will be excreted [51]. With this view in mind, a pharmacokinetic study of the ligand molecules was carried out using an in silico approach. The drug-like properties of the test ligands were evaluated using MolSoft chemoinformatics software. Lipinski’s rule states that a drug is likely to have good absorption and permeation if the candidate molecules have: (1) molecular weight < 500, (2) Log P < 5, (3) number of hydrogen atom donors < 5, and if (4) hydrogen atom acceptors (N and O) are <10. The logP values recorded for all the ligands were in the range of 2.28 to 6.10 (Table 3). All the test compounds satisfied Lipinski’s rule, except kurchessine and conessine, which showed one violation (Table 3). The control drug loperamide, also did not obey Lipinski’s rule [49]. The MolLogP value observed for loperamide was slightly higher than the recommended value of 5. It is worth mentioning here that loperamide is a synthetic opioid-like agent that is not significantly absorbed from the gut [52]. However, the compliance with Lipinski’s rule of five in the of case of the test ligands, suggests their favorable pharmacological properties.

### 2.7. ADMET Prediction

The properties of all the ligands with respect to their prediction of absorption, distribution, metabolism, excretion, and toxicity were evaluated by admetSAR online tool (http://lmmd.ecust.edu.cn:8000/) The advantage of using this in silico approach is that it can reduce the attrition rate of the drugs to a great extent [53]. The results predicted by admetSAR revealed that all the ligand molecules, including loperamide, had a positive HIA score (Table 4). The positive HIA score is indicative of the better bioavailability of the drug. Oral bioavailability is considered an important parameter for the development of bioactive molecules, as therapeutic agents and Caco-2 cell permeability are used as a reliable in vitro/in silico model to predict oral drug absorption [54]. The results presented in Table 4 demonstrate that all the test ligands could penetrate through the Caco-2 cells, except holadysenterine. It has been suggested that lipophilicity, hydrogen bond donor (HBD), and polar surface area (PSA) are the key factors that regulate the cell permeability of a drug. The predicted values of polar surface area (PSA) for the test ligands, conessine, kurchessine, isoconessimine, pubescine, holadienine, holanamine, conessimine, holadysenterine, kurchine, and loperamide, were 6.03, 5.76, 15.09, 50.49, 16.93, 39.05, 14.99, 74.72, 15.09, and 34.51 Å^2^, respectively (Table 3). Lazerwith et al. (2011) [55] reported that the PSA value of a compound has an inverse relationship with its lipid permeation capability. If the polar surface area (PSA) value of a drug is greater than 140 Å^2^, and HBA > 10, HBD > 5, and MW > 500, in that case it is quite likely that it (the drug) will have a limited cell membrane permeability [56]. On the contrary, a compound with a PSA value <75 Å^2^, combined with a high lipophilicity (logP > 4) can have an increased risk of adverse events [57]. This was the case observed for kurchessine, conessine, isoconessimine, holadienine, conessimine, kurchine, and loperamide, where the PSA values were 5.76, 6.03, 15.09, 16.93, 14.99, 15.09 Å, 34.51, the and LogP values were 6.10, 5.16, 4.61, 4.25,4.67, 4.61, and 5.39, respectively (Table 3). Veber et al. (2002) [58] suggested that for good oral bioavailability, the PSA value of a compound should not exceed 120–140 A^2^. From this discussion, pubescine, holadysenterine, and holanamine emerge as the compounds of choice.

The polar surface area (PSA) has also been used as a predictor for blood–brain barrier (BBB) penetration by many investigators [59]. Compounds with a strong hydrogen bond forming potential have less penetration through the BBB. In the current study, all the test compounds, including the control drug loperamide, showed positive results for BBB penetration (Table 4). Though loperamide is a non-absorbable drug, a minute quantity of it can be detected in systemic circulation when taken at the recommended dose, and at higher doses it has the ability to cross the blood brain barrier [52]. This finding is in tune with our data (Table 4).

The orally administered drug, after reaching the gastrointestinal tract, permeates through the biological membrane to enter the systemic circulation. It can cross the intestinal epithelium either passively or by active transport. Active transport is mediated by transport proteins such as ATP binding P- glycoprotein (P-gp). It functions as an efflux pump and exports a large number of drugs from cells, resulting in reduced intestinal absorption and enhanced elimination into bile and urine [60]. This indicates that P-gp has a great impact on the ADME properties of a variety of drugs [61,62]. Therefore, it was decided to examine whether or not the test ligands were substrates for P-gp. The results, described in Table 4a, revealed that kurchessine, conessine, isoconessimine, pubescine, holadienine, conessimine, kurchine, and the control drug loperamide were substrates and inhibitors of P-gp. On the other hand, holanamine and holadysenterine were found to be substrates and non-inhibitors of P-glycoprotein. 

Cytochrome P450 (CYP450), a superfamily of isoforms, has been shown to play a key role in the oxidative and reductive metabolic transformation of drugs used in clinical practices. Of all the CYP enzymes, CYP3A4 is the most abundant enzyme in the liver and is used by more than 50% of drugs for their metabolism and elimination [63,64]. Drug metabolism via CYP enzymes causes several clinically relevant drug–drug interactions, which ultimately may lead to a variety of adverse drug reactions and drug toxicity etc. [65]. In this context, a number of drugs have been identified as substrates, inhibitors, and inducers of CYP enzymes. The results presented in (Table 5) showed that all the ligands, including the control drug-loperamide, were substrates and non-inhibitors of CYP3A4. On the other hand, holadysenterine was found to be a substrate and inhibitor of CYP3A4 (Table 5). The inhibition of CYP3A4 suggests a strong possibility of drug interactions with other CYP3A4 metabolized co-administered drugs, which may cause accumulation of the drug at a concentration greater than the acceptable limit [66,67]. However, adjustment of the dose of CYP3A4 inhibitor during co-administration with other CYP3A4 substrates could help to maintain an appropriate level of the drug [65]. 

The term acute toxicity means the adverse effects of a drug observed after its exposure within a short period of time. This is aimed at assessing the safety of a drug and is normally performed during the first stage of toxicological investigation [68,69]. All the test ligands were evaluated by AMES toxicity test, carcinogenicity test, and rat acute toxicity test. All the ligands, including the control drug loperamide, gave negative test result in the AMES toxicity test (Table 6). This indicates that the test compounds are not mutagenic. Comparing the LD50 doses obtained for each ligand in the rat model, they were found to be in an acceptable range. In our study, loperamide had the highest dose of 3.65 mol/kg (Table 6). Among the test ligands, pubescine displayed the highest LD50 value of 2.92 mol/kg, followed by holadysenterine with a LD50 value of 2.49 mol/kg. Holanamine had the lowest LD50 value of 2.19 mol/kg, which is in an acceptable range (Table 6).

### 2.8. Molecular Dynamics Analysis

The dynamic nature and protein–ligand stability of the three lead compounds were analyzed by a molecular dynamics study [70].

**RMSD and RMSF analysis of holanamine and ECD complex**

**Figure 6:**
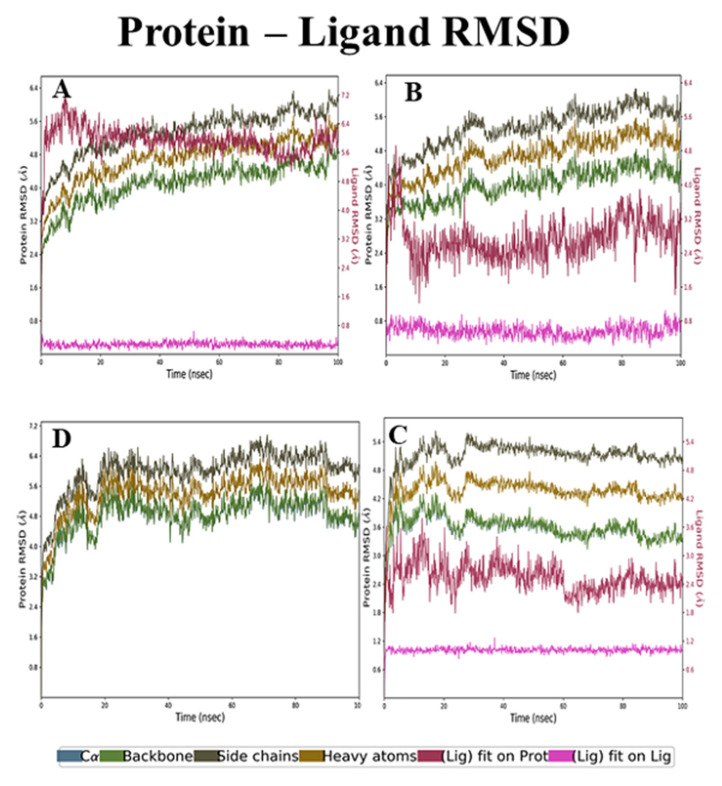
RMSD analysis of lead compounds and ECD_GC-C_ protein complexes. (**A**) Holanamine, (**B**) Holadysenterine, (**C**) Pubescine, (**D**) No ligand.

**Figure 7:**
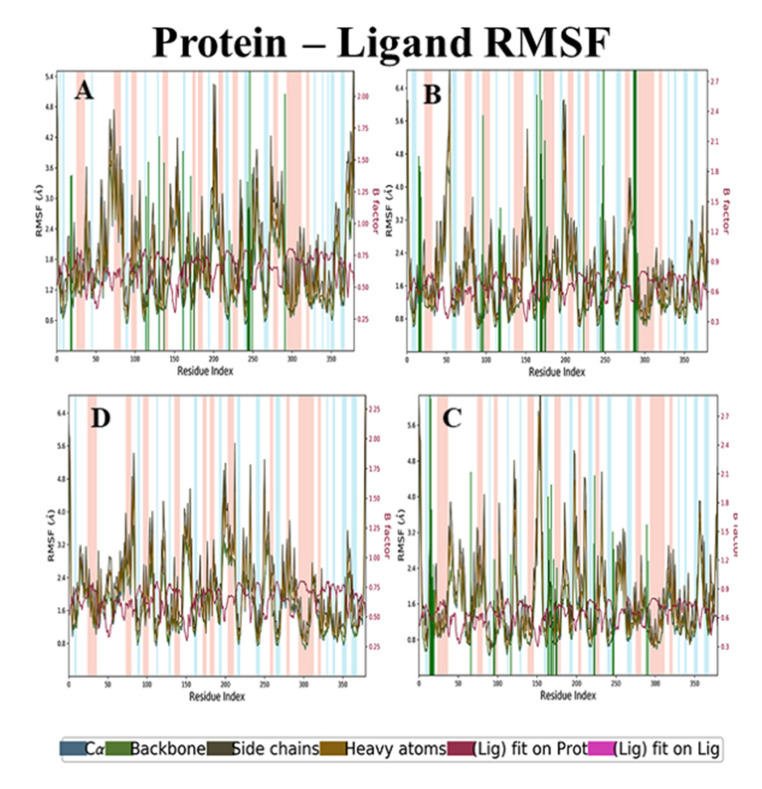
Residue RMSF analysis of lead compounds and ECD_GC-C_ protein complexes. (**A**) Holanamine, (**B**) Holadysenterine, (**C**) Pubescine, (**D**) No ligand. The background color denotes helix (light pink) and loop (sky blue) regions of the protein.

The backbone RMSD analyses of holanamine and ECD complex were maintained at 2.4 to 4.0 Å. The side chain RMSD started from 3.2 Å and ended at 4.8 Å. The RMSD of heavy atoms was 4.0 to 6.0 Å. The ligfit on protein initially had a light deviation and attained stability after a 40-ns time interval with 4.8 Å (Figure 6A). The RMSF analysis revealed that the non-active residue regions 75–80, 200–220, and 275–280 had slightly higher fluctuations (4 to 4.8 Å) in the backbone, heavy atoms, and side-chain, but the ligand fit showed a smaller fluctuation, from 1.6 to 1.8 Å (Figure 7A). The RMSD and RMSF analyses of holanamine and ECD complex, despite its less deviation compared to apoprotein, maintained stability throughout the simulation. 

**RMSD and RMSF analysis of holadysenterine and ECD complex**

The RMSD of backbone, side-chain, and heavy atoms had 3.2 to 3.5 Å, 4.0 to 4.5 Å, and 4.8 to 5.3 Å, respectively. The results (Figure 6B) further revealed that the backbone, side-chain, and heavy atoms did not show a high deviation and maintained stability throughout the simulation. The ligfit graph initially had slight fluctuations but after 10 ns it attained stability at 2.4 Å, which was comparatively lesser deviation than that of holanamine and ECD complex. The RMSF analysis of backbone, side-chain, and heavy atoms, despite the non-active residue regions 45–55, 145–155, 200–220, and 275–280, had a higher fluctuation (Figure 7B). The ligfit graph was maintained at 1.5 Å throughout the simulation period. The RMSD and RMSF analyses revealed that the holadysenterine and ECD complex were similar to apoprotein and ligand, and well-bound to the protein throughout the simulation.

**RMSD and RMSF Analyses of Pubescine–ECD Complex**

In the case of pubescine–ECD complex, the RMSD analysis of ligfit was maintained at 2.4 Å in a 100-ns time interval. The RMSD of backbone, side-chain, and heavy atoms had 3.2 to 3.5 Å, 4.0 to 4.5 Å, and 4.8 5.3 Å, respectively (Figure 6C). The RMSD analysis revealed that the pubescine–ECD complex was similar to apoprotein, holadysenterine, and ECD complex. The RMSF analysis of backbone, side-chain, and heavy atoms, despite the non-active residue regions 45–55, 145–155, 200–220, and 275–280, had higher fluctuations (Figure 7C). The ligfit graph was at maintained 1.2 Å throughout the simulation period. The results show that the pubescine–ECD complex maintained stability and had less fluctuations at the 100-ns time interval.

**Figure 8:**
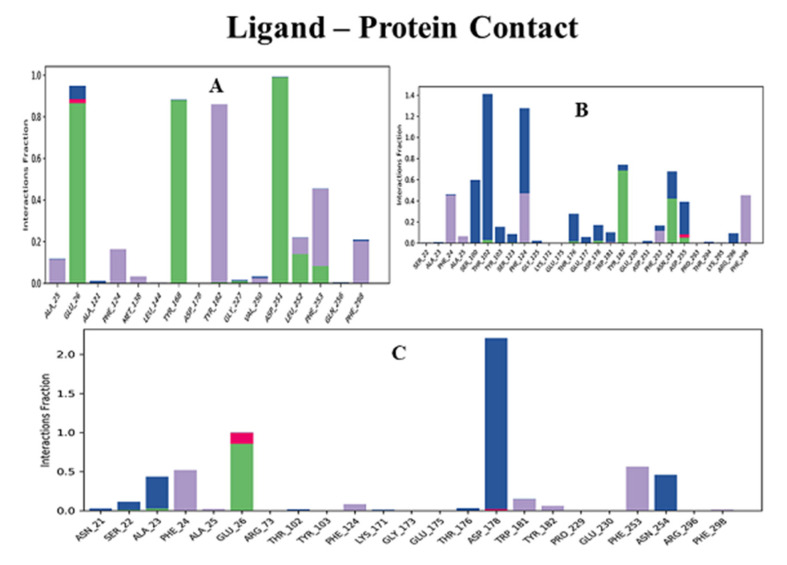
Hydrogen bond contact analysis of lead compounds and ECD_GC-C_ protein complexes. Various intermolecular interactions made by ECD pocket amino acid residues with lead ligands during molecular dynamics simulations. (**A**) Holanamine, (**B**) Holadysenterine, (**C**) Pubescine. Bar colors: Hydrogen bond (Green), Hydrophobic (Purple), Ionic (Red), Water bridge (Blue).

Furthermore, the protein–ligand contact (Figure 8) showed that Glu26, Tyr102, Phe124, Tyr168, Asp178, Tyr182, Asp251, and Asn254 residues of ECD made hydrogen bond contacts with the ligands throughout the simulation time. The overall results of the molecular dynamics showed that all three compounds were stable and interacted with the protein during the simulation period. These results were very well correlated with the results of the molecular docking.

### 2.9. Molecular Interaction of Ligands with Amino Acids of the Target Protein

**Figure 9:**
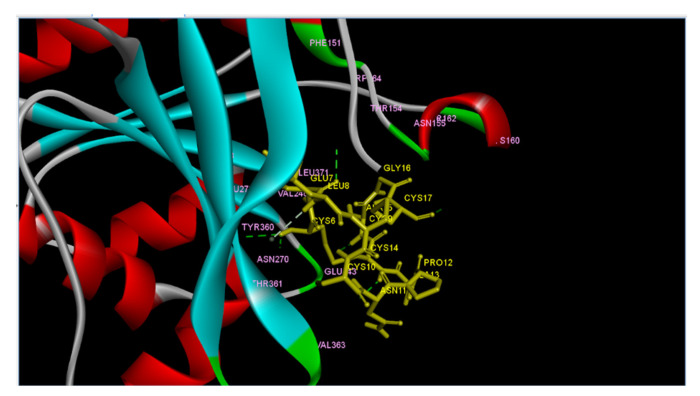
Model 0 of ECD_GC-C_ and STa (PDB ID-1ETN) returned by ClusPro and visualized by Discovery Studio Visualizer. ECD is represented as ribbons. 1ETN is shown in tubular form. H-bonds are represented as dashed lines in a green color.

Furthermore, we wanted to find out if the lead compounds and STa share the same binding site in terms of amino acid residues on ECD. This required the identification of the amino acid residues on ECD interacting and binding with STa. It is worth mentioning here that Wada et al. (1996) [35], using site directed mutagenesis, showed ARG136 and ASP347 to be amino acid residues binding to STa in the extracellular domain of pigStaR. They also suggested that a region from ASP347 to Val 401, close to the transmembrane domain, is crucial for STa binding activity and guanylyl cyclase catalytic activity. Hasegawa et al. (1999) [71] designed a photoaffinity labelled analog of STa and used it for the identification of the ligand binding site on the extracellular surface of GC-C. They reported the ligand binding region between 387 to residue 393 on ECD. In the present study we attempted to investigate the binding of STa on a modelled structure of ECD using an in silico approach. We performed the docking of STa against ECD using ClusPro 2.0. The output of the study was in the form of three cluster centers. The binding affinity in ClusPro is determined by cluster size and not in scores or probability [72,73]. The cluster 0 (zero), having the maximum number of members (865 members), was selected and the model was visualized through Discovery Studio Visualizer (Figure 9). We discovered from this experiment that ECD formed six hydrogen bonds with STa (Table 7). The amino acid residues of the extracellular domain (ECD) engaged in hydrogen bond formation with STa were THR154, LYS160, GLU243, ASN270, and TYR360 (Table 7). The next question we addressed was to investigate if the lead compounds also bind to the same amino acid residues of ECD as STa. This was done by comparing the results obtained from two different docking approaches, viz protein–protein docking (Figure 9) and molecular docking (Figure 5). Our molecular docking studies (Figure 5) demonstrated that the lead compounds holanamine and pubescine did not show binding affinity for the amino acid residues of ECD which formed hydrogen bonds with STa (Figure 9). On the other hand, holdysenterine, the second-best compound, formed a hydrogen bond with ASN270. It also made pi-alkyl and pi-sigma interactions with the TYR360 and THR154 of ECD. Our results in Table 7 show that the ASN270 of ECD forms hydrogen bonds with the CYS6 of STa. In addition, TYR360 and THR154 also form hydrogen bonds with the CYS6 and GLU7 of STa (Table 7). These results suggest the strong affinity of holadysenterine for ASN270, TYR360, and THR154 will possibly weaken/inhibit interactions between STa and ECD.

## 3. Materials and Methods

### 3.1. Plant Material

The plant sample (stem bark) of *Holarrhena pubescens* Wall. ex G. Don was collected during October 2019 from the forest area of Chitrakoot in Satna District of Madhya Pradesh, India. The latitude and longitude of Chitrakoot are 25.1043° N and 80.5155° E. It has dry climate. The township experiences maximum temperature of 49 degree Celsius in the month of May and minimum of 5 degree Celsius in winters. The forest of the Chitrakoot is predominantly tropical dry mixed deciduous type. The plant sample was identified by Dr. R. L. S. Sikarwar (Plant Taxonomist) who was working as Head of the Division of Medicinal Plants Garden, Arogyadham, Deendayal Research Institute, Chitrakoot, Satna, Madhaya Pradesh, India. The voucher specimen (No. 29) was deposited in the herbarium section, Division of Medicinal Plants Garden of the aforesaid institute. The stem bark sample after collection was shade dried, ground to a coarse powder, and stored in airtight containers for further use.

### 3.2. Test Strain

The bacterial culture of enterotoxigenic *E. coli* (ETEC) in the present study was a gift from Dr. Manisha Yadav Dhanda, Ambedkar Centre of Biomedical Sciences, University of Delhi, Delhi, India [74]. The bacterial cultures were inoculated separately on a nutrient agar plate and incubated at 37 °C for 24 h in an incubator.

### 3.3. Extraction and Preparation of Alkaloid Rich Fraction

The dry powdered stem bark (500 g, in portions of 5 × 100 g) was extracted in dichloromethane and an alkaloid enriched fraction was prepared, as described by Nnadi et al. (2017) [75].

### 3.4. Antimicrobial Activity

The antimicrobial activity of plant extracts was measured using a standard disc diffusion assay, by measuring the zone of inhibition against the test organisms [76]. Enterotoxigenic *E. coli* (ETEC) used in this study was a clinical isolate, and 0.1 mL of diluted test organism (0.5 Mcfarland standard) was spread on Muller Hinton agar (HiMedia) plates. Sterile filter paper discs of 6 mm were placed on the prepared agar plate. Initially, the antimicrobial activity of alkaloid fraction was studied at a concentration of 100 mg/mL. Minimum inhibitory concentration was determined by loading 10 µL of the alkaloid fraction at various concentrations (100 mg/mL, 50 mg/mL, 25 mg/mL, 12.5 mg/mL, 6.25 mg/mL, 3.125 mg/mL) on separate individual discs. Then, 10 μL sterile water was pipetted on one of the discs to serve as a negative control, and for solvent control 10 μL of acetone was added on a separate disc. In addition to this, 10 μL of 10 µg gentamycin was loaded on another disc and used as a positive control. The plates were kept at room temperature for a period of 1 h for diffusion and then incubated for 24 h at 37 °C. The zone of inhibition was measured and compared with the positive and negative controls. Each experiment was repeated in five replicates. The results were expressed as average of the zones of inhibition.

### 3.5. Protein Model Generation

The extracellular domain of guanylyl cyclase c (ECD_GCC_) expressed in intestinal epithelial cells was selected as a receptor for the docking study. The amino acid sequence of guanylyl cyclase c of *Homo sapiens* was obtained from Universal Protein Resource (UniProt ID: P25092). Since the 3D structure of the receptor guanylyl cyclase c was not available in the Protein Data Bank database, the structure was modeled by homology modeling approach using Swiss Model workspace (http://swissmodel.expasy.org) [33]. 

The modeled structure was verified using PROCHECK [40], ERRAT [41], and Verify3D (UCLA DOE) [36]. The active site of the ECD was determined using the CASTp3.0 server.

### 3.6. Docking Studies

Selection of the major alkaloids from *Holarrhena pubescens* Wall.ex G. Don (kutaj) was made on the basis of their antidiarrheal activity reported in the literature [32]. The nine major ligands selected in this study were kurchessine, conessine, isoconessimine, pubesciene, holadienine, holanamine, conessimine, holadysenterine, and kurchine. Besides these, loperamide, an antidiarrheal drug, was used as a control sample. All the selected ligands were docked to the active site of ECD using AutoDock 4.2 [44]. A grid box was set at 120 × 120 × 120 to provide enough space for the free movement of the ligands and to control the docking site. For each ligand, ten different poses were generated and the top-ranking pose was selected for further study. The protein ligand interaction was analyzed using Discovery Studio Visualizer software package (Dassault Systèmes, San Diego, CA, USA) [45].

### 3.7. Ligand and Protein Preparation

The structures of the ligands were sketched using ChemSketch Package version ACD/Labs 2017.12 (https://www.acdlabs.com/resources/freware/chemsketch/, accessed on September 2018) and then converted into pdb format by OpenBabel Package version 2.4.1 (http://openbabel.org) which was accessed on September 2018. The ligands in PDBQT format were derived from PDB format using AutoDock 4.2. The structures of the ligands and protein were optimized for docking.

The Lamarckian genetic algorithm was applied to model the interaction pattern between the receptor protein and ligands.

### 3.8. Drug-Likeness Prediction

The molecular descriptors, such as partition coefficient (LogP), polar surface area (PSA), hydrogen bond donors and acceptors, number of atoms, molecular weight were computed using the MolSoft online tool (www.molsoft.com/mprop/, accessed on 14 November 2019). Using these parameters, the ligands were evaluated for their compliance with Lipinski’s rule of five [49].

### 3.9. ADMET Screening

The parameters of absorption, distribution, metabolism, excretion, and toxicity describe the ADMET properties of a compound. In the present study the ADMET profile of the test ligands was predicted using the admetSAR online tool (http://lmmd.ecust.edu.cn:8000/, accessed on 19 December 2019).

### 3.10. Molecular Dynamics Simulation

The stability of the protein–ligand complex was analyzed using the Desmond package [77]. The OPLS3 force field and TIP3P water model were used for simulation. Furthermore, it was neutralized by adding Na^+^/Cl^−^ ions. Energy minimization steps were carried out using a steepest descent method (50,000 steps), and NVT and NPT ensembles were also performed for 100 and 300 ps, respectively [70]. The long-range electrostatic interactions in the system were calculated by the Particle Mesh Ewald (PME) algorithm [78]. Final MD simulations (100 ns) were performed for each complex.

### 3.11. Protein–Protein Docking

ClusPRO software package version 2.0 (https://cluspro.bu.edu/, accessed on December 2018) [72,73] was used for protein–protein docking. The crystal structure of heat stable enterotoxin (STa) (PDB ID.1ETN) was taken from the RCSB Protein Data Bank and was used as a ligand. The 3D model of the extracellular domain (ECD) of guanylyl cyclase c generated by Swiss Model workspace [33], as discussed in Model Generation (2.2), was used as a receptor for the docking experiments.

## 4. Conclusions

The therapeutic importance of alkaloids in the treatment of diarrhea and dysentery has been reported in literature. Based on this information the current study was designed aiming to discover ligands capable of inhibiting/interfering with the binding of STa on ECD_GC-C_. Our disc diffusion assay, conducted to evaluate the antibacterial activity of the alkaloid rich fraction of *Holarrhena pubescens* against ETEC, demonstrated very encouraging results. By the screening of nine steroidal alkaloid ligand types from *H. pubescens* for their binding affinity towards ECD_GC-C_, we identified three ligands. These compounds were in close association with the target protein and possessed good drug-like properties, as shown by the molecular dynamics simulations and in silico ADMET prediction, respectively. The experiments to identify the ability of these leads to interfere with the binding of STa on ECD_GC-C_ were carried out in two steps. In the first step amino acid residues of ECD binding to STa, in terms of hydrogen bonds, were recognized by protein–protein docking. The second step involved the identification of amino acid residues of target protein, which formed hydrogen bonds with the lead compounds in the docking experiment. These amino acid residues were matched with the amino acid residues from first step. Our results showed that out of the three best hits, holadysenterine formed hydrogen bonds with ASN270 of ECD. The same amino acid also took part in the binding to STa and formed hydrogen bonds with CYS6 of STa. We also observed that the drug made pi-alkyl and pi-sigma interactions with the TYR360 and THR154 of ECD. These amino acid residues were also seen to form hydrogen bonds with the CYS6 and GLU7 of STa. The results presented here are based on preliminary experiments and require further validation involving in vitro assays and experiments in animal models. This is the first study reporting that holadysenterine has the required qualities to be a potent antidiarrheal drug against ETEC induced diarrhea. 

## Figures and Tables

**Table 1:** (**A**): Mean diameter of inhibition zones (mm) for *E. coli* (ETEC) growth inhibited by alkaloid rich fraction of *Holarrhena pubescens* (kutaj). (**B**): Disc diffusion test for antimicrobial activity of *Holarrhena pubescens* (kutaj): (**a**) Zone of inhibition of positive control (gentamycin), (**b**) Zone of inhibition of alkaloid fraction, (**c**) Zone of inhibition of negative control.

Enterotoxigenic *E. coli* (ETEC)				
A				B
Treatment	Concentration	Dose/Disc	Zone of Inhibition	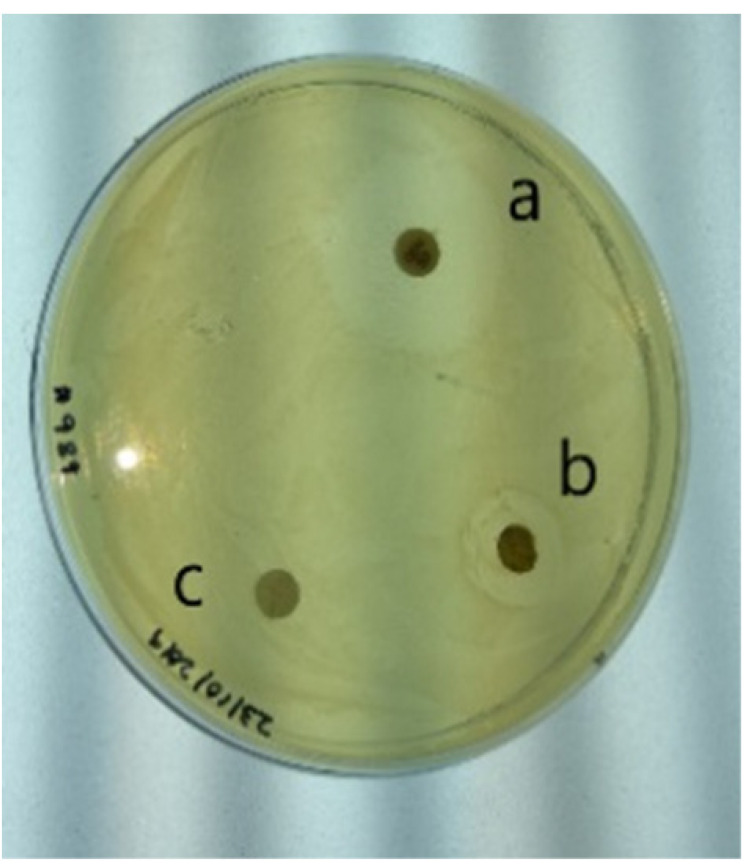
Alkaloid Rich Fraction (mg/mL)	100 mg/mL	1 mg	16 ± 0.38 mm	
	50 mg/mL	0.5 mg	14 ± 0.53 mm	
	25 mg/mL	0.25 mg	0.0 ± 0.0	
	12.5 mg/mL	0.125 mg	0.00 ± 0.0	
	6.25 mg/mL	0.625 mg	0.00 ± 0.0	
Positive control (Gentamycin)		10 µg	35 ± 0.707 mm	
Negative Control		-	Nil	
Solvent Control		-	Nil	

**Table 2:** Ligands used for docking with ECD. **^a^** Binding energy values of the test ligands, **^b^** number of hydrogen bonds formed with the ligands.

Ligand	PubChem ID	Binding Energy ^a^ kcal/mol	H Bond ^b^	Structure
Pubescine	72313	−8.05	2	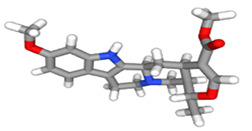
Kurchessine	442979	−8.52	NA	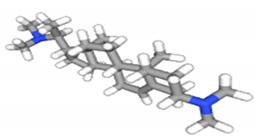
Holadienine	12310532	−8.54	NA	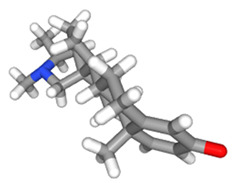
Conessimine	12303831	−8.59	NA	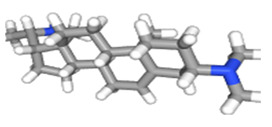
Conessine	441082	−9.00	NA	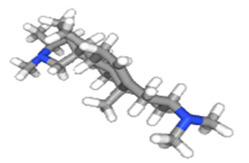
Holadysenterine	16742955	−8.06	5	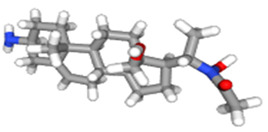
Isoconessimine	11772257	−9.05	NA	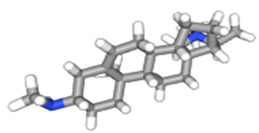
Kurchine	551434	−9.05	NA	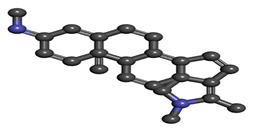
Holanamine	6869-29-0	−8.44	1	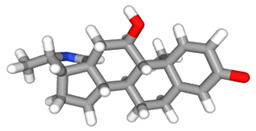
Loperamide	3955	−8.05	NA	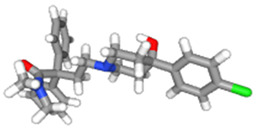

**Table 3:** Physicochemical parameters of the ligands.

Ligand	Mol wt.	No. of HBA	No. of HBD	MolLogP	MolPSA
Kurchessine	372.35	2	0	6.10	5.76 Å^2^
Conessine	356.32	2	0	5.16	6.03 Å^2^
Isoconessimine	342.30	2	1	4.61	15.09 Å^2^
Pubescine	382.19	5	1	2.28	50.49 Å^2^
Holadienine	325.24	2	0	4.25	16.93 Å^2^
Holanamine	325.20	3	1	3.37	39.05 Å^2^
Conessimine	342.30	2	1	4.67	14.99 Å^2^
Holadysenterine	390.29	4	4	2.59	74.72 Å^2^
Kurchine	342.30	2	1	4.61	15.09 Å^2^
Loperamide	476.22	3	1	5.39	34.51 Å^2^

**Table 4:** ADMET Properties of the Ligands.

Ligand	Blood Brain Barrier	Caco-2 Permeability	Human Intestinal Absorption	P-Glycoprotein Substrate
Kurchessine	BBB+	Caco2+	HIA+	Substrate, Inhibitor
Conessine	BBB+	Caco2+	HIA+	Substrate, Inhibitor
Isoconessimine	BBB+	Caco2+	HIA+	Substrate, Inhibitor
Pubescine	BBB+	Caco2+	HIA+	Substrate, Inhibitor
Holadienine	BBB+	Caco2+	HIA+	Substrate, Inhibitor
Holanamine	BBB+	Caco2+	HIA+	Substrate, Non-inhibitor
Conessimine	BBB+	Caco2+	HIA+	Substrate, Inhibitor
Holadysenterine	BBB+	Caco2-	HIA+	Substrate, Non-inhibitor
Kurchine	BBB+	Caco2+	HIA+	Substrate, Inhibitor
Loperamide	BBB+	Caco2+	HIA+	Substrate, Inhibitor

**Table 5:** ADMET Properties of the Ligands (Metabolism).

Ligand.	CYP2C9 Substrate	CYP2D6 Substrate	CYP4503 A4 Substrate	CYP450 1A2 Inhibitor	CYP4502C9 Inhibitor	CYP4502D6 Inhibitor	CYP450 3A4 Inhibitor
Kurchessine	Non substrate	Non-Substrate	Substrate	Non-inhibitor	Non-inhibitor	Non inhibitor	Non inhibitor
Conessine	Non substrate	Non Substrate	Substrate	Non inhibitor	Non inhibitor	Non inhibitor	Non inhibitor
Isoconessimine	Non substrate	Non substrate	Substrate	Non-inhibitor	Non-inhibitor	Non inhibitor	Non inhibitor
Pubescine	Non substrate	Non substrate	Substrate	Inhibitor	Non inhibitor	Inhibitor	Non inhibitor
Holadienine	Non substrate	Non substrate	Substrate	Non inhibitor	Non inhibitor	Non inhibitor	Non inhibitor
Holanamine	Non substrate	Non substrate	Substrate	Non inhibitor	Non inhibitor	Non inhibitor	Non inhibitor
Conessimine	Non substrate	Non Substrate	Substrate	Non inhibitor	Non inhibitor	Non inhibitor	Non inhibitor
Holadysenterine	Non substrate	Non substrate	Substrate	Non inhibitor	Non inhibitor	Non inhibitor	Inhibitor
Kurchine	Non substrate	Non Substrate	Substrate	Non inhibitor	Non inhibitor	Non inhibitor	Non inhibitor
Loperamide	Non substrate	Non substrate	Substrate	Non inhibitor	Non inhibitor	Inhibitor	Non inhibitor

**Table 6:** ADMET Predicted Profile (Toxicity).

Ligand	AMES toxicity	Carcinogenicity	Rat Acute Toxicity (mol/kg)
Kurchessine	Non AMES toxic	Non-carcinogens	2.5575
Conessine	Non AMES toxic	Non-carcinogens	2.6198
Isoconessimine	Non AMES toxic	Non-carcinogens	2.6781
Pubescine	Non AMES toxic	Non-carcinogens	2.9255
Holadienine	Non AMES toxic	Non-carcinogens	2.5022
Holanamine	Non AMES toxic	Non-carcinogens	2.1900
Conessimine	Non AMES toxic	Non-carcinogens	2.6437
Holadysenterine	Non AMES toxic	Non carcinogens	2.4973
Kurchine	Non AMES toxic	Non carcinogens	2.6781
Loperamide	Non AMES toxic	Non carcinogens	3.6560

**Table 7:** List of the amino acid residues forming hydrogen bonds in a protein–protein interface for ECD and Heat stable Enterotoxin STa (PDB ID.1ETN).

S.No	Heatstable Enterotoxin STa	Extacellular Domain ECD	Bond Length (Å)
1-	ALA15	GLU243	2.09
2-	CYS6	ASN270	2.87
3-	CYS6	TYR360	2.01
4-	GLU7	THR154	2.51
5-	CYS14	GLU243	2.22
6-	CYS17	LYS160	1.68

## Data Availability

Data available on request.

## Abbreviations

ADMET	Absorption: distribution, metabolism, excretion and toxicity
BBB	Blood brain barrier
CFTR	Cystic fibrosis transmembrane conductance regulator
ECD	Extracellular domain
ECD_GC-C_	Extracellular domain of Guanylyl cyclase c
ETEC	Enterotoxigenic *Escherichia coli*
GC-C	Guanylyl cyclase c
GCs	Guanylyl cyclases
HBA	Hydrogen bond acceptor
HBD	Hydrogen bond donor
HIA	Human Intestinal Absorption
IBD	Inflammatory bowel disease
IBS	Irritable bowel syndrome
MLCK	Myosin light chain kinase
NHE3	Na^+^/H^+^ exchanger isotype 3
NPR-A	atrial brain natriuretic peptide
NPR-B	brain natriuretic peptide
NPR-C	Natriuretic Peptide Receptor-C
PDB	Protein Data Bank
P-gp	P- glycoprotein
PSA	Polar surface area
RCSB	Research Collaboratory for Structural Bioinformatics
RMSD	Root mean square deviation
RMSF	Root mean square fluctuation
STa	Heats stable enterotoxin
TJ	Tight junction

## Sample Availability

Not available.
